# Cleansing Formulations That Respect Skin Barrier Integrity

**DOI:** 10.1155/2012/495917

**Published:** 2012-08-13

**Authors:** Russel M. Walters, Guangru Mao, Euen T. Gunn, Sidney Hornby

**Affiliations:** ^1^Johnson & Johnson Consumer Products Companies, 199 Grandview Road, Skillman, NJ 08558, USA; ^2^Neutrogena Corporation, 5760 West 96th Street, R&D Building, Los Angeles, CA 90045, USA

## Abstract

Surfactants in skin cleansers interact with the skin in several manners. In addition to the desired benefit of providing skin hygiene, surfactants also extract skin components during cleansing and remain in the stratum corneum (SC) after rinsing. These side effects disrupt SC structure and degrade its barrier properties. Recent applications of vibrational spectroscopy and two-photon microscopy in skin research have provided molecular-level information to facilitate our understanding of the interaction between skin and surfactant. In the arena of commercial skin cleansers, technologies have been developed to produce cleansers that both cleanse and respect skin barrier. The main approach is to minimize surfactant interaction with skin through altering its solution properties. Recently, hydrophobically modified polymers (HMPs) have been introduced to create skin compatible cleansing systems. At the presence of HMP, surfactants assemble into larger, more stable structures. These structures are less likely to penetrate the skin, thereby resulting in less aggressive cleansers and the integrity of the skin barrier is maintained. In this paper, we reviewed our recent findings on surfactant and SC interactions at molecular level and provided an overview of the HM technology for developing cleansers that respect skin barrier.

## 1. Introduction and History

The general purpose for skin cleansing is to reduce sebum and exogenous contaminants and to control odors and the skin microbiome. The surfactants in cleansers solubilize hydrophobic materials into the aqueous phase and enable their subsequent removal from the skin surface. The amphiphilic structure of surfactants, consisting of both a hydrophilic polar head group and a nonpolar lipophilic tail, drives surfactants to oil/water interfaces to facilitate cleansing. 

**Figure 1:**
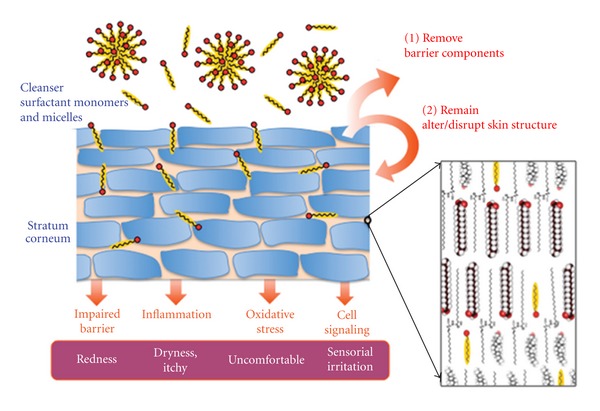
Depiction of how surfactants within a cleanser can remove SC material and also remain in the SC. The lengths scales of the cartoon at the left are inaccurate; corneocytes have diameters ~20 *μ*m, while the micelles sizes are ~5 nm. At the right, a molecular-level illustration of ordered SC lipids (ceramides, cholesterol, and fatty acids) and surfactants from a cleanser inserting into these ordered SC lipids.

Figure 1 depicts how the surfactants interact with the stratum corneum (SC) during cleansing. Cleansers are usually formulated with surfactant at concentration much higher than its critical micelle concentration (CMC). At such concentration, the majority of the surfactant molecules self-assemble into micelles [1]. It is desirable for a cleanser to remove unwanted exogenous lipophilic materials; however, the interaction between surfactants and skin is more complicated. Solubilization of skin components such as lipids, enzymes, and natural moisturizing factors weakens the skin barrier function. Additionally surfactants can also remain in the SC even after rinsing and lead to chronic surfactant exposure [2]. SC structure is composed of anucleated corneocytes embedded in an intercellular lipid matrix. These lipids form a highly ordered lamellar structure [3]. As will be discussed later, surfactant molecules that remain in the SC likely insert into the SC lipid lamellae, which is schematically graphed in the inset of Figure 1. The inserted surfactants disrupt SC lipid structural order and cause the continual degradation of the skin barrier [4, 5]. As the result of the barrier impairment, inflammation and oxidative stress occur [6, 7], which can then be perceived by patients as redness, dryness, discomfort, and irritation of the skin.

**Figure 2:**
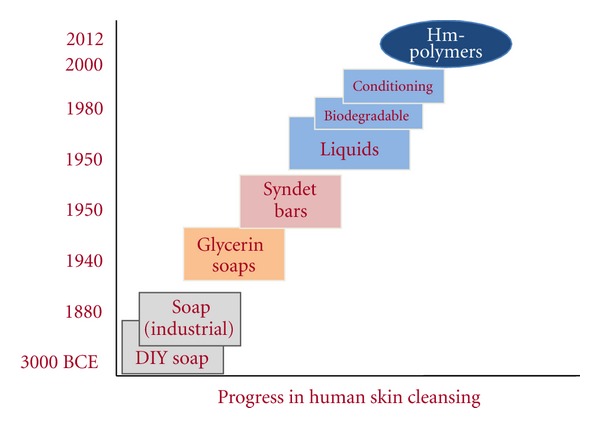
Progress of technology and skin compatibility of human skin cleansing over time. Adapted from Walters 2009.

Humans have been cleansing their skin with surfactants for millennia. Soap was discovered multiple times throughout human history.  Figure 2 shows the technical progression of skin cleansing over time. Generally, the progress in cleansing technology has been marked by the creation of cleansing systems that better respect the skin barrier. While the industrial revolution brought purer soap, the high pH and aggressiveness that came with this new product motivated the development of new, gentler technologies [8, 9]. The addition of glycerin to cleansers, to make for milder cleansing systems, marked the first significant advance in skin cleansing. 

After the world wars, the development of new synthetic chemistries enabled many advances in milder cleansing. Developed in the 1950s, the lower pH syndet bar was introduced as an alternative cleanser to soap. The syndet bar has been shown to better respect the skin barrier than soap bars [10]. In the 1960s, polymers were added to cleansers for the first time for multiple benefits [11]. As will be discussed later, the more recent introduction of hydrophobically modified polymers into surfactant systems allows for a new approach to creating cleansers with reduced impact to the skin barrier. 

## 2. Surfactant Penetration into Skin

In order to design a cleansing formulation that respects the skin barrier, it is essential to understand how surfactant penetrates into skin. It has long been believed that only surfactant monomers can penetrate into skin [12, 13], which is known as “surfactant monomer skin penetration model.” This model was largely based on the observations that surfactant-induced irritation is positively correlated with the CMC of surfactant mixtures and the CMC is the upper limit of monomer concentration in a solution. In addition, micelles were generally believed not able to penetrate into skin due to their larger size. 

The monomer penetration theory drove a desire to decrease the CMC of cleansing systems, which led to the development of surfactant systems with low monomer concentrations or low critical micelles concentrations (CMCs), that were believed to be less irritating [14]. Personal care cleansers are primarily comprised of anionic surfactants (commonly sodium lauryl sulfate), and adding a cosurfactant has reliably reduced their CMCs and lowered the aggressiveness of cleansers to the skin barrier. 

More recent discoveries have challenged monomer penetration model to fully explain how surfactants penetrate skin. Following surfactant exposure, skin irritation and barrier disruption increases with increasing concentration of surfactant, even at surfactant concentrations above the CMC, where the monomer level is constant [15–17]. Additionally, typical dermal exposure occurs at concentrations of 1–10 wt% surfactant, concentrations that are two or three orders of magnitude above the CMC concentrations. At these typical in-use concentrations nearly all of the surfactants exist in micelles with only a small fraction ~0.1% existing as monomers. Finally, the correlation between surfactant CMCs (the monomer level) and aggressiveness was not found in systems studied more recently [18]. 

Researchers have proposed alternative mechanisms to explain these discrepancies of monomer penetration model. Blankschtein and associates utilized radiolabeled ^14^C to track the amount of SDS penetrated into epidermis and found it to increase with SDS concentration when applied above the CMC [19]. When polyethylene oxide (PEO) was added to the SDS solution, less SDS was observed in the epidermis. PEO primarily interacts with micelles but not with monomers. The PEO-bound SDS micelles have an average radius of 25 Å, while that of the unbound micelles is ~20 Å. It was suggested that SDS micelles, with its small size, could be capable of penetrating the skin through aqueous pores, while the larger PEO-bound SDS micelles could not and a surfactant micelle skin penetration model was proposed. While the current research is actively evaluating this micelle model, it has already inspired new technologies to think outside of the box of CMC-based cleanser design approach.

## 3. Effects of Surfactants on Skin at Molecular Level

SC, the outermost layer of the skin, provides most of the skin's barrier function. As discussed previously, it is structured as stratified anucleated corneocytes embedded in an intercellular lipid matrix [20], which is mainly composed of ceramides, long-chain free fatty acids, cholesterol, and cholesterol sulfate [21–24]. SC lipids are organized as multiple lamellae with long and short periodicity [25–30]. In each lamella, the lipids are laterally packed in predominantly orthorhombic and hexagonal phases [31]. Such highly ordered SC lipid structures play an important role in regulating water transport and skin permeability [32, 33]. The disruptions of the SC lipid order by surfactants contribute to the barrier damaging side effects of skin cleansing [34, 35]. Our group and our collaborators have recently studied sodium dodecyl sulfate (SDS) penetration in both isolated SC [36] and excised intact skin [37] with infrared spectroscopy and confocal Raman microscopy to understand the effects of SDS on skin structure at molecular level and the time course of its permeation in skin.

**Figure 3:**
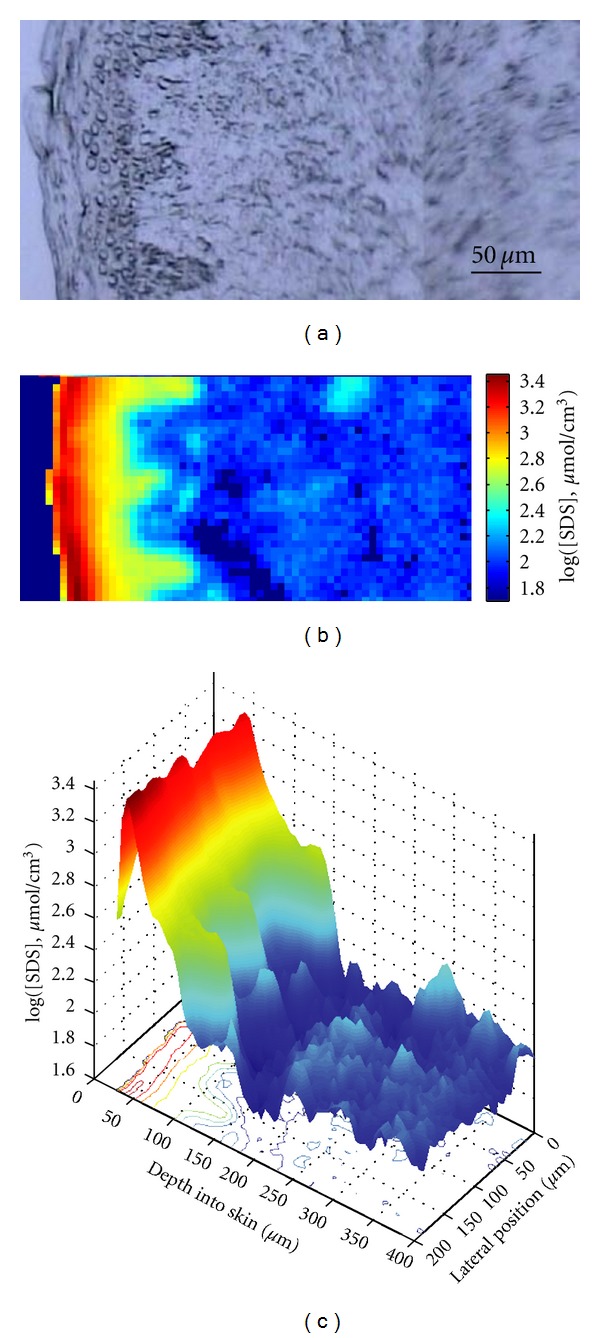
(a) Visible light microscopic image of human skin cross-section, distribution of SDS concentration in the same skin section following 40 h topical SDS treatment at 34°C, (b) shown as an IR image map, and (c) shown as a 2D depth profile.

In these studies, acyl chain perdeuterated SDS was utilized to accomplish the simultaneous detection of IR and Raman signals originating from both permeated SDS and endogenous skin lipids and proteins. For experiments conducted with isolated SC, the amount of SDS that permeated into SC became saturated after 2 h SDS soaking. It took longer time for topically applied SDS to permeate into the full thickness skin. Distribution of the absolute SDS concentration in skin cross-section was determined through IR spectroscopic imaging technique with ~10 *μ*m spatial resolution and 5–20% accuracy in concentration measurement. SDS permeated into different skin regions in a time- and temperature-dependent manner. SDS concentration up to 1000 mmol/L, which is much higher than donor solution (40 mmol/L), was observed in SC. The results for a skin sample treated with SDS for 40 h at 34°C are shown in Figure 3, along with the companion microscopic image. The rapid SDS concentration decrease going from SC into viable epidermis demonstrates the barrier function of SC. SDS was observed to permeate into the dermis region at a concentration of ~32 mmol/L. 

**Figure 4:**
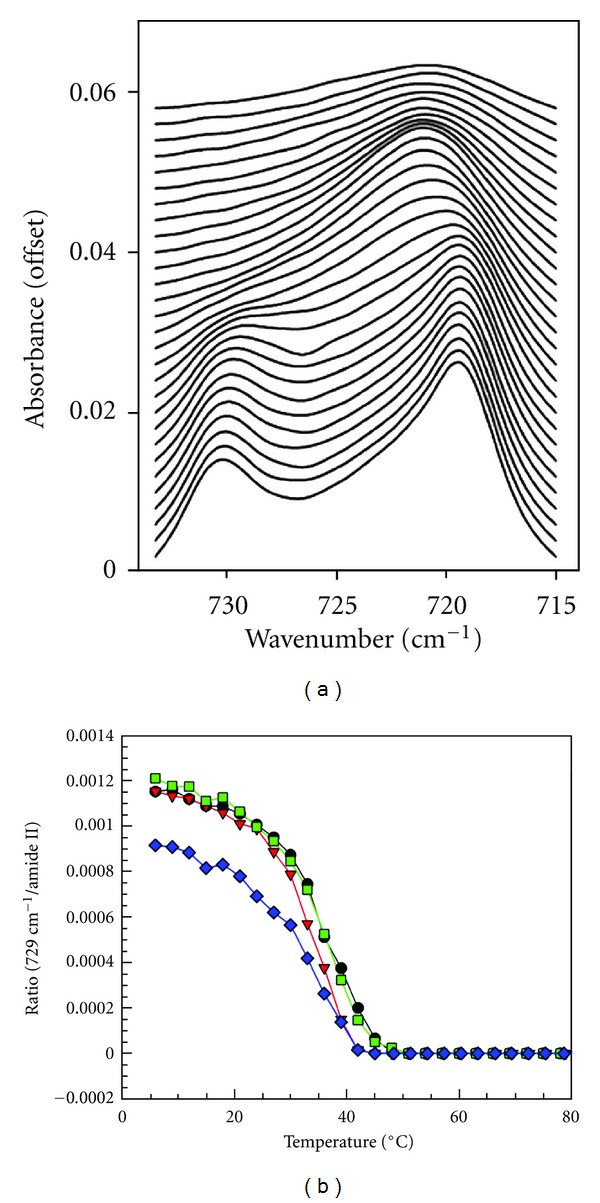
(a) CH_2_ rocking band contour progression with temperature increase from 6°C (bottom) to 90°C (top) in 3°C increments a in an isolated human SC control sample; (b) integrated peak area of 729 cm^−1^ rocking band normalized by protein Amide II peak area as a function of temperature for 2 h control (circles line), 2 h SDS-d_25_ (down-pointing triangles line), 6 h control (square line), and 6 h SDS-d_25_ (rhombuses line) isolated human SC samples.

In addition to tracking SDS penetration, IR spectroscopy offers a convenient approach to evaluate the interaction between surfactants and skin by following skin lipid order and protein secondary structure as well as the physical state of permeated SDS molecules. A set of spectra between 715 and 732 cm^−1^ from isolated SC is plotted in Figure 4 as a function of temperature. The methylene rocking band in this spectral region is sensitive to phase transition between orthorhombically (ortho) and hexagonally (hex) packed lipids. At low temperature, human SC lipids are mainly packed in orthorhombic phase and display two peaks near 729 cm^−1^ and 720 cm^−1^. As temperature increases, the amount of lipids in hexagonal phase increases, and, as a result, the 729 cm^−1^ peak intensity diminishes and 720 cm^−1^ peak red shifts slightly. Therefore, the 729 cm^−1^ peak is utilized as a signature of orthorhombic phase. Its integrated peak area, normalized by protein Amide II peak area to account for SC thickness difference between samples, is depicted in Figure 4(b) as a function of temperature. After isolated SC was soaked with SDS, the midpoint of this ortho-to-hex phase transition temperature decreases and the initial amount of SC in orthorhombic phase was lower compared to controls. SDS appears to be extracting lipids and/or increasing the amount of hexagonal phase or disordering lipids that were originally in orthorhombic phase. 

**Figure 5:**
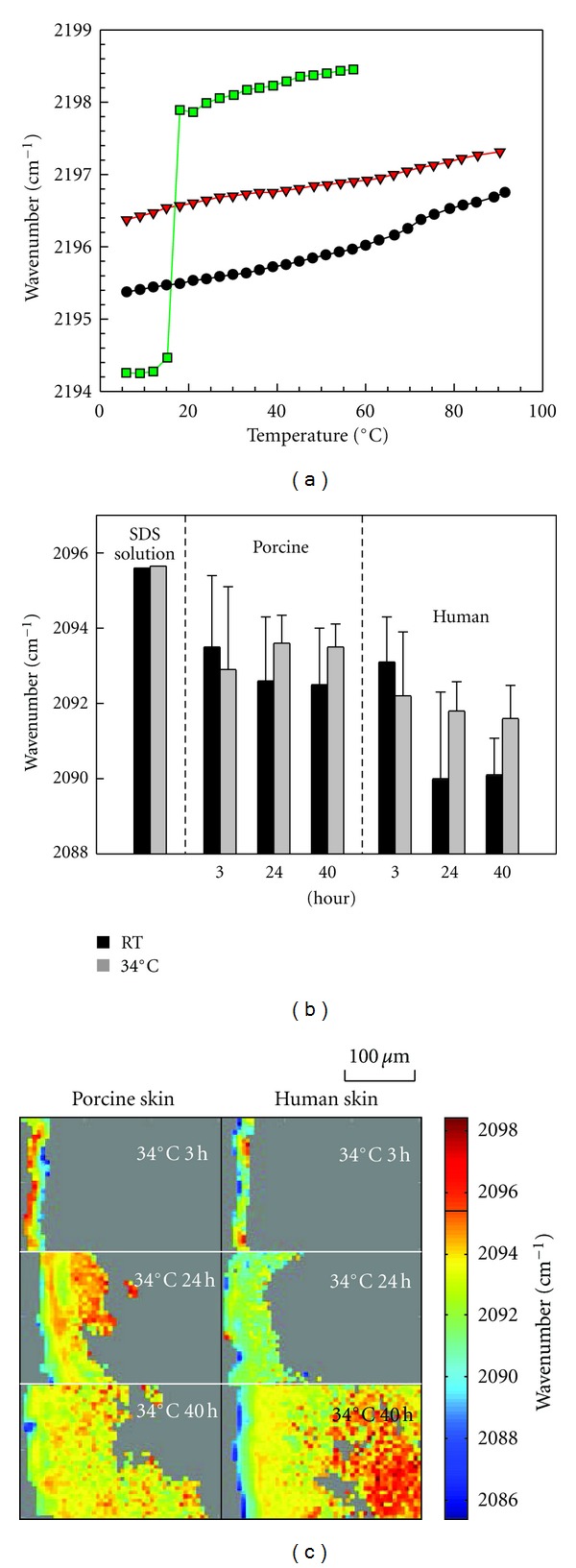
(a) Peak frequency of the SDS-d_25_ CD_2_ asymmetric stretching band as a function of temperature in isolated SC after 2 h (circles line) and 6 h (squares line) SDS-d_25_ incubation along with a SDS-d_25_ solution in PBS at 62.5 mg/mL (down-pointing triangles line); (b) average peak frequency of SDS-d_25_ CD_2_ symmetric stretching in porcine and human SC after 3, 24, and 40 h treatment at room temperature (gray) and 34°C (black) along with a SDS-d_25_ solution in PBS at 12.5 mg/mL. Error bars (standard deviation) do not reflect lack of precision in the measurement but rather predominantly arise from heterogeneity in the skin; (c) peak frequency of SDS-d_25_ CD_2_ symmetric stretching frequency in porcine and human skin after treatment for 3, 24, and 40 h at 34°C.

SDS conformational order can be tracked with methylene stretching frequency, the lower the frequency is the more ordered the acyl chains are.  Figure 5(a) shows the asymmetric stretching frequency of SDS in micelles and in SC after 2 h and 6 h soaking as a function of temperature. The frequency increase from 2194 cm^−1^ to 2198 cm^−1^ at ~18°C for SDS solution corresponds to its Krafft point, above which SDS is predominantly in a micellar phase. As shown in the figure, when incorporated to isolated SC, SDS asymmetric frequency was ~1.5–3 cm^−1^ lower comparing to its micellar state. Similar decrease in stretching frequency comparing to SDS micelles was also observed for SDS permeated into the SC regions of full thickness skin. The symmetric methylene stretching frequency between 2090 and 2096 cm^−1^ was monitored for SDS in the SC of intact skin and is shown in Figure 5(b). The decrease in stretching frequency and thus increase in conformational order for SDS in SC indicate that SDS exists in a more ordered state in SC than SDS micelles. The densely packed SC lipids apparently have an ordering effect on permeated SDS. For the SDS that penetrated to the deeper dermis sites of skin, its stretching frequency is comparable to micelles (Figure 5(c)). These observations provide some insights into the mode of SDS permeation in skin. SDS can either permeate into skin as a monomer or permeate as micelles but these micelles quickly dissemble to monomers once integrated into SC lipids. The possibility of micelle reformation in dermis is not likely but cannot be excluded based on the above CMC concentration in these sites and the stretching frequency comparable to SDS micellar solution. 

Protein secondary structure is commonly monitored with Amide I and Amide II band contours between 1480 and 1730 cm^−1^. The lack of major changes in this spectral region for isolated SC and SC from full thickness skin before and after SDS treatment demonstrates that SDS has minimal effects on SC keratin structure. The ability of surfactants to solubilize zein protein has been used to access the surfactant harshness. However, it might not be relevant to the actual interaction between surfactant and SC proteins. Zein protein is structured as antiparallel helices clustered within a distorted cylinder [38], while the SC keratin has a more complicated secondary structure and assembles to keratin filaments [39]. Furthermore, the keratin inside cornified envelope of SC is much more difficult to access compared to the zein protein in testing solutions. The surfactant permeates into SC mainly through intercellular lipid pathway and might have minimal contact with keratin inside corneocyte envelope. This hypothesis is consistent with a recent study on naturally fluorescent penetration enhancers [40]. The two-photon fluorescence microscopy images of skin treated with a more hydrophobic molecule, sodium sulforhodamine G (SRG), showed that SRG is mostly confined in the cornified envelope and did not penetrate inside the corneocytes.

Increases in transepidermal water loss (TEWL) following SDS treatment have been reported [6, 34, 41, 42]. In addition to damaging the skin barrier, SDS permeation causes irritation and inflammation [7, 43] and alters barrier renewing processes by affecting keratinocyte differentiation [44] and desquamation [45]. The disordering effects of SDS on SC lipids help explain the weakened skin barrier and offer a mechanism for the observed TEWL increases following SDS treatment. The fact that it is able to permeate to deep sites in skin can be responsible for the irritation and inflammation that are commonly associated with SDS application on skin.

## 4. Creating Cleansers with Less Barrier Disruption

As discussed, surfactants are capable of disrupting the skin barrier, and creating cleansing formulations with minimal barrier disruption has marked the major advancement in cleansing technologies. By modifying their solution properties, the behavior of the surfactants can be changed, and the effect of surfactants on the skin barrier can be reduced. In addition to the CMC, surfactant solution properties including the surface charge, size, and shape of micelles, as well as the dynamics of the surfactant monomer-micelle equilibrium, are major factors to consider when designing the new generation of skin cleansers. 

Surfactant micelles that have a highly negative surface charge (i.e., micelles of anionic surfactants) have been shown to be more aggressive at solubilizing Zein protein [46]. By blending amphoteric surfactants, the micelle surface charge is reduced, and the surfactant system becomes less aggressive. Modifying the aqueous phase can also affect the surfactant behavior. For instance, Ghosh et al. demonstrated that adding glycerin to SDS solution leads to reduced barrier perturbation when compared to SDS control [5, 47].

In an alternate approach, polymers have been used to alter surfactant solution behavior in order to create milder cleansers. Polyethylene oxide (PEO) has been shown to alter micelles and create surfactant systems with less aggressiveness to the skin barrier [17]. The PEO chains bind water molecules and have been shown to wrap around surfactant micelles [48]. These polymer chains with bound water are highly biocompatible, as they present water to biological tissue. This approach to mild cleansing actually was employed decades ago; the original mild cleansing technology in baby shampoo was employed in PEG-80 Sorbitan Laurate to create mild cleansing systems [49, 50].

More recently, alternate polymer architectures have been used to modify surfactant solution behavior. Hydrophobically modified polymers (HMPs) have been shown to associate surfactants in solution. Surfactant self-assembled to the hydrophobic domains of the polymer results in slower surfactant dynamics. By creating these large polymer/surfactant complexes, the cleanser becomes less aggressive [51]. In these HMP/surfactant systems, because less surfactant enters the SC, there is less inflammation, and therefore the skin barrier is less disturbed [52]. 

In recent work, we have developed a gentle foaming facial cleanser utilizing HMP. The effects on the skin barrier following treatment with this formulation (NUG) compared to a leading dermatologist-recommended lotion facial cleanser (CGSC) were compared. With images obtained from multiphoton laser scanning confocal microscope [53], the benefits provided by HMP technology of minimizing the SC barrier disruption were visualized directly. The skin samples were mounted on a Franz diffusion cell with SC facing the donor chamber and cleansers, diluted with distilled water to a concentration of 80%, were applied and maintained at 37°C for 2 h. A fluorescent dye was then applied to the samples, and its fluorescence in skin was imaged. The penetration of this florescent dye characterized the barrier properties of skin samples treated with different cleansers [54]. 

**Figure 6:**
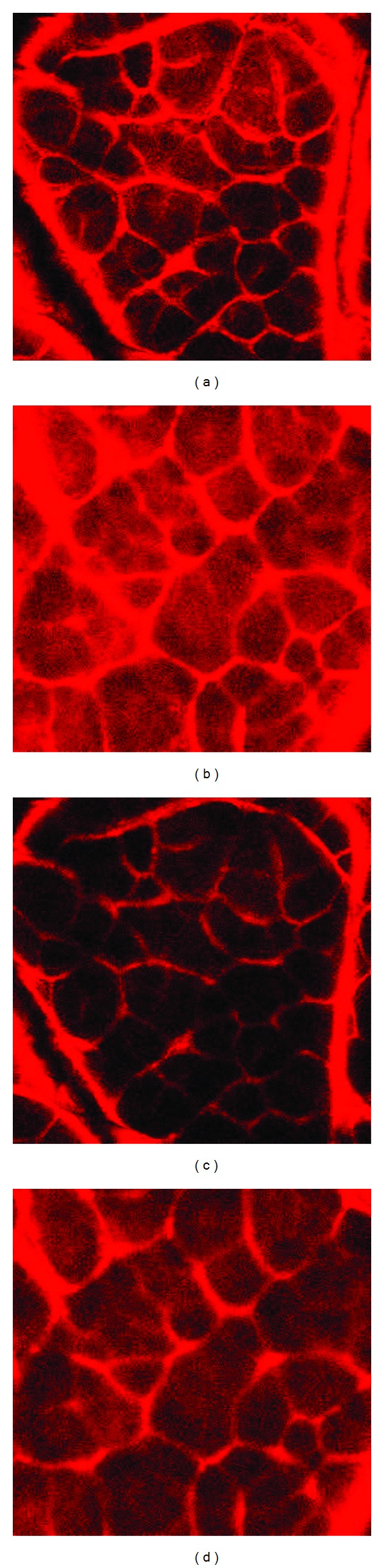
2-photon fluorescent microscopy images showing skin barrier condition after treatment with NUG facial cleanser, with HMP (a and c) compared to CGSC (b and d) at a depth into the SC of 2 *μ*m (upper row; a and b) and 20 *μ*m (lower row; c and d). The limited dye penetration (lower intensity) indicates a more intact barrier, while more dye penetration indicates a weaker barrier.

Typical photomicrographs of the dye penetration in skin samples after exposure to two cleansers are shown in Figure 6. The images from depths into the skin at 2 and 20 *μ*m are shown in the top and bottom rows, respectively. Lower intensity of fluorescence indicates a more intact barrier after exposure to the cleansing system, while higher dye penetration signifies a more porous barrier. With both cleansers, images obtained at the 20 *μ*m skin depth, Figures 6(c) and 6(d), show less presence of dye compared to the ones from 2 *μ*m depth into skin, Figures 6(a) and 6(b). Comparing images obtained from skin treated with different cleansers, at the same skin depth, the image from skin treated with NUG clearly had less fluorescence from the dye than that from CGSC treated skin (Figure 6(a) versus Figure 6(b), and Figure 6(c) versus Figure 6(d)). The lower intensities of the dye in the NUG-treated specimens demonstrated the reduced barrier damage caused by cleanser compared to CGSC. 

## 5. Conclusions 

Surfactants remove skin components, penetrate into skin, alter skin structure, and therefore degrade skin barrier functions and lead to clinical and subclinical skin conditions. Maintaining the molecular order of the SC lipids is essential to healthy skin. The new understanding of the interactions between SDS, which has entered the SC, and the SC lipids at molecular level reveals the importance of designing cleansing systems that respect skin barrier function.

In order to maintain the skin barrier during cleansing, it is best to maintain the endogenous lipids and the native skin structure. The addition of polymeric species that interact with the surfactants to modern cleanser formulations creates less aggressive cleansers. The novel application of hydrophobically modified polymers has been proven to advance current technology to further minimize the damaging effects of cleansers on skin.
